# East Mediterranean Lineage of *Brucella melitensis* in Human Isolates and Milk Samples in Oman Using MLVA-14

**DOI:** 10.12688/f1000research.161111.1

**Published:** 2025-02-12

**Authors:** Khalsa Altoubi, Zakariya Al Muharrmi, Waleed AlMarzooqi, Salma Al Adwani, Kaadhia Al Kharousi, Shytyrbayeva Zamzagul Abdildaevna, Simone Peletto, Yasmin ElTahir

**Affiliations:** 1 Central Laboratory for Animal Health, Department of Animal Diseases Diagnosis, Oman, Muscat, Oman; 2 Animal & Veterinary Sciences, Sultan Qaboos University, Muscat, Muscat Governorate, Oman; 3 Microbiology & Immunology, Sultan Qaboos University, Muscat, Muscat Governorate, Oman; 4 Biological Safety, Kazakh National Agrarian Research University, Almaty, Kazakhstan; 5 Istituto Zooprofilattico Sperimentale del Piemonte, Turin, Italy

**Keywords:** Brucella melitensis, MLVA-14, genetic diversity, Oman.

## Abstract

**Background:**

Brucellosis is the most common zoonotic disease in Oman. Studies about genetic diversity of *Brucella* are limited in the country. This study aimed to genotype *Brucella melitensis* in human isolates and milk samples using multi-locus variable number tandem repeats analysis (MLVA-14) in Oman.

**Methods:**

MLVA-14 was employed for forty-nine *B. melitensis* recovered from human isolates (n = 21), one goat isolate, and milk samples (n = 27).

**Results:**

Clustering analysis separated the 49 *B. melitensis* strains into two main clusters including 31 genotypes. In Dhofar Governorate, shared genotypes among different animal species were identified; the same genotypes were found also in human isolates. Moreover, there was a close genetic relationship between human and milk sample strains from Dhofar and AD Dakhiliya Governorates. Phylogeography investigated by Minimum Spanning Tree analysis showed that Omani strains belonged to the East Mediterranean lineage and formed a distinct branch with a close relationship with two strains from the United Arab Emirates. Moreover, eight Omani strains genotyped from milk shared the same MLVA profile as strains from Spain, Portugal, China, India, and Turkey. The caprine isolate was an outlier correlated with a big cluster mostly formed by isolates from China with other strains from Portugal, Kazakhstan, Turkey, Mongolia, Marocco, France and Spain.

**Conclusions:**

This study highlights the zoonotic nature of *B. melitensis* transmission from infected livestock to humans and also its circulation among different animal species. The One Health approach is the way to develop policies and programs for disease surveillance and control.

## Introduction

Brucellosis is a zoonotic disease affecting human and animal species, including livestock, wild animals, and marine mammals. In animals, it causes abortion, mastitis, reproductive disorders, and reduced milk production. In human, brucellosis can cause various symptoms, from a mild flu-like illness to severe complications such as arthritis and endocarditis, making early detection and control critical. The disease is considered as a main public health concern due to its impact on livestock productivity and human health. ^1– 3^

In Oman, brucellosis remains the most common zoonosis. The disease is endemic in Dhofar Governorate due to its humid climate, especially during the monsoon season. A common practice of keeping animals, such as cattle, sheep, and goats, in proximity significantly increases the chances of *Brucella* spillover. Moreover, human-to-animal contact increases the risk of transmission, necessitating robust surveillance and control measures. ^4– 6^

*Brucella* is an intracellular Gram-negative coccobacillus bacterium. There are various *Brucella* species. The classic known six species are *B. abortus*, *B. melitensis*, *B. ovis*, *B. canis*, *B. suis*, and *B. neotomae.* ^7^ More species were recognized later like, *B. ceti*, *B. pinnipedialis*, *B. microti*, *B. inopinata*, *B. papionis*, and *B. vulpis.* ^8– 10^ *Brucella melitensis* has been widely reported in humans and livestock in Oman. ^4, 6^

*Brucella* can be isolated by culture and identified by serological tests and molecular assays. ^11^ It can also be identified according to CO _2_ requirements and biochemical tests such as H _2_S production, urease activity, and agglutination with monospecific sera (A and M). Further identification can be achieved using selective media containing dyes such as thionin or basic fuchsin and phage typing. *Brucella* isolation is the most reliable method for diagnosis but it is time-consuming and needs special facilities. ^12^

Rose Bengal plate test is a rapid test for screening *Brucella* antibodies in both livestock and humans. ^13^ A milk ring test is utilized to identify *Brucella* antibodies in cow’s milk. ^14^ Moreover, another screening test is the *Brucella* rapid test which can be used for various field samples including blood, plasma, serum, and milk. ^15^ Enzyme-linked immunosorbent Assays (ELISA) have been widely used as a confirmatory test. ^16^

The *Brucella* genome encodes virulence factors and metabolic pathways crucial for intracellular survival. *Brucella* species show similarity higher than 90% at the genome level, making it difficult to differentiate between *Brucella* strains through conventional genetic analysis techniques. ^17– 19^

Molecular methods are widely used for *Brucella* diagnosis such as conventional PCR, real-time PCR, multiplex PCR, multiple loci variable number tandem repeat analysis (MLVA), and single nucleotide polymorphism (SNP) analysis. Conventional PCR involves DNA amplification of a single target (singleplex PCR) or multiple targets (multiplex PCR). AMOS and Bruce-ladder are multiplex PCR methods that amplify multiple *Brucella* target genes in a single reaction. These assays use multiple sets of primers, each specific to a different region in the genome, to differentiate between *Brucella* species. ^20– 23^

MLVA is widely used to identify *Brucella* genetic diversity. A tandem repeat is a sequence of two or more DNA base pairs repeated and directly adjacent to each other in the genome. These repeats can vary in length and number. MLVA can be used in various genetic studies, including genetic fingerprinting, studying genetic diversity, and identifying hereditary diseases. By analyzing tandem repeats within the genome, MLVA enables high-resolution differentiation of strains, and offering valuable insights into the genetic makeup of *Brucella.* ^22, 24^ The faster accumulation of genetic variation in tandem repeat markers compared to SNP-based variation, allows genotyping and distinguishing between closely related *Brucella* strains. ^25^ MLVA technique is cost-effective and serves as an excellent alternative to other sequencing methods. ^22^

Due to the zoonotic nature of brucellosis, tracing infection sources, identifying transmission pathways, and implementing effective control strategies are crucial for epidemiologic surveillance in Oman. Accurately distinguishing between *Brucella* species and biovars is important for treatment, controlling the disease, and developing of a common vaccine. DNA-based genotyping methods are preferred, as they offer greater discriminatory power compared to phenotypic methods. This study aimed to investigate the genetic makeup of *Brucella* strains circulating in different geographical locations in Oman in both human and livestock using MLVA.

## Methods

### Ethical considerations

This study was approved by the Medical Research Ethics Committee (MERC) in the College of Medicine & Health Sciences at Sultan Qaboos University (SQU) REF. NO. SQU-EC/060/2023 for using human samples. The research project submitted to the Medical Research Ethics Committee (MREC), College of Medicine and Health Sciences, Sultan Qaboos University for re-consideration and approval was discussed. The modifications received by the Committee on 3rd May 2023 in response to the comments raised during the 30th March 2023 meeting were found to be satisfactory. The Committee has considered the research project acceptable and therefore approval was granted. For animal samples, there was no need for the Sultan Qaboos University ethical committee’s approval as the study did not involve experimental research on animals. The samples were used after verbal consent from the patients without disclosing their names.

The animals were needed only for milk samples. The milking was done in a clean quiet area to ensure a peaceful environment for the animals. There was minimal handling and restraint under the consent, help, and guidance of the owners who are very familiar with their stock. Milking was carried out aseptically by experienced technicians. The clinician was around to ensure the safety of the animals during the procedure. This asserts that animal welfare was not compromised in any way more than minimal normal handling for aseptic milking under clinical supervision in the presence of animal owners.

One hundred and sixteen milk samples from different animal species and twenty-one human blood samples were collected from Dhofar and AD Dakhiliyah governorates in Oman. Brucella in human blood samples was identified by incubating blood in an aerobic blood medium vial. Then, samples were incubated in the BD BACTEC™ blood culture system. After that, the samples were streaked directly into the blood and chocolate agar media simultaneously and incubated for 24-48 hours. Gram staining and biochemical tests (urease and oxidase) were performed for confirmation.

Milk samples were screened for *Brucella* presence using a milk ring and rapid tests. Then, seropositive samples were incubated for 24 hours at 37°C in a shaker incubator. Subsequently, samples were centrifuged, the supernatant was removed, and 200 μL of phosphate buffer saline (PBS) was added to the pellet. DNA extraction was done for all samples as previously described by Yu & Morrison. ^26^ To confirm the presence of *Brucella* genome in milk samples, real-time PCR was carried out using the BruSpp dtec-qPCR kit (Genetic Analysis Strategies, Spain) followed by conventional PCR based on three *Brucella*-specific primer pairs as previously described. ^27– 29^ Moreover, species-specific primers (Bruce-ladder) were employed, as previously described. ^21^

MLVA genotyping of *B. melitensis* was carried out using the *BRUCELLA* MLVA-16 Typing Kit (Genoscreen, France), where 16 markers are amplified from purified DNA using four quadruplex PCR reactions; the refined set of 16 VNTR was previously outlined. ^7, 30^ MLVA-16 analysis was performed on 21 human isolates, a goat isolate, and 42 DNA extracted from milk samples. Specifically for milk samples, eight markers (Bruce 04, 11, 45, 30, 21, 43, 42, and 19) were amplified through singleplex PCR. Each PCR was conducted under identical parameters to ensure consistency across samples. The PCR products from the singleplex reactions were pooled into two multiplex groups before undergoing capillary electrophoresis. Capillary electrophoresis and fragment analysis of PCR products were conducted by two companies: GenoScreen (France) and Macrogen (South Korea). All PCR products were run on an ABI 3730XL capillary sequencer ^44^ utilizing POP7 polymer to ensure high-resolution fragment separation. Two size standards were applied based on the service provider. GeneScan 600LIZ size standard (Applied Biosystems) was used for samples analyzed by macrogen. GeneScan 1200LIZ size standard (Applied Biosystems) was used for samples analyzed by GenoScreen.

### Data analysis

Data of *Brucella* strain genotypes of this study were compared with MLVA genotypes of the *Brucella* MLVA website ( http://mlva.u-psud.fr/brucella/) in November 2024. Band size estimates were translated into a number of units using BioNumerics version 7.6 (Applied Maths, Belgium). For clustering analyses (dendrogram), the categorical coefficient and the unweighted-pair group method using average linkages (UPGMA) algorithm were used. Minimum Spanning Trees (MST) were constructed using BioNumerics applying categorical coefficients together with the single and double locus variance priority rules.

## Results

**Figure 1.**
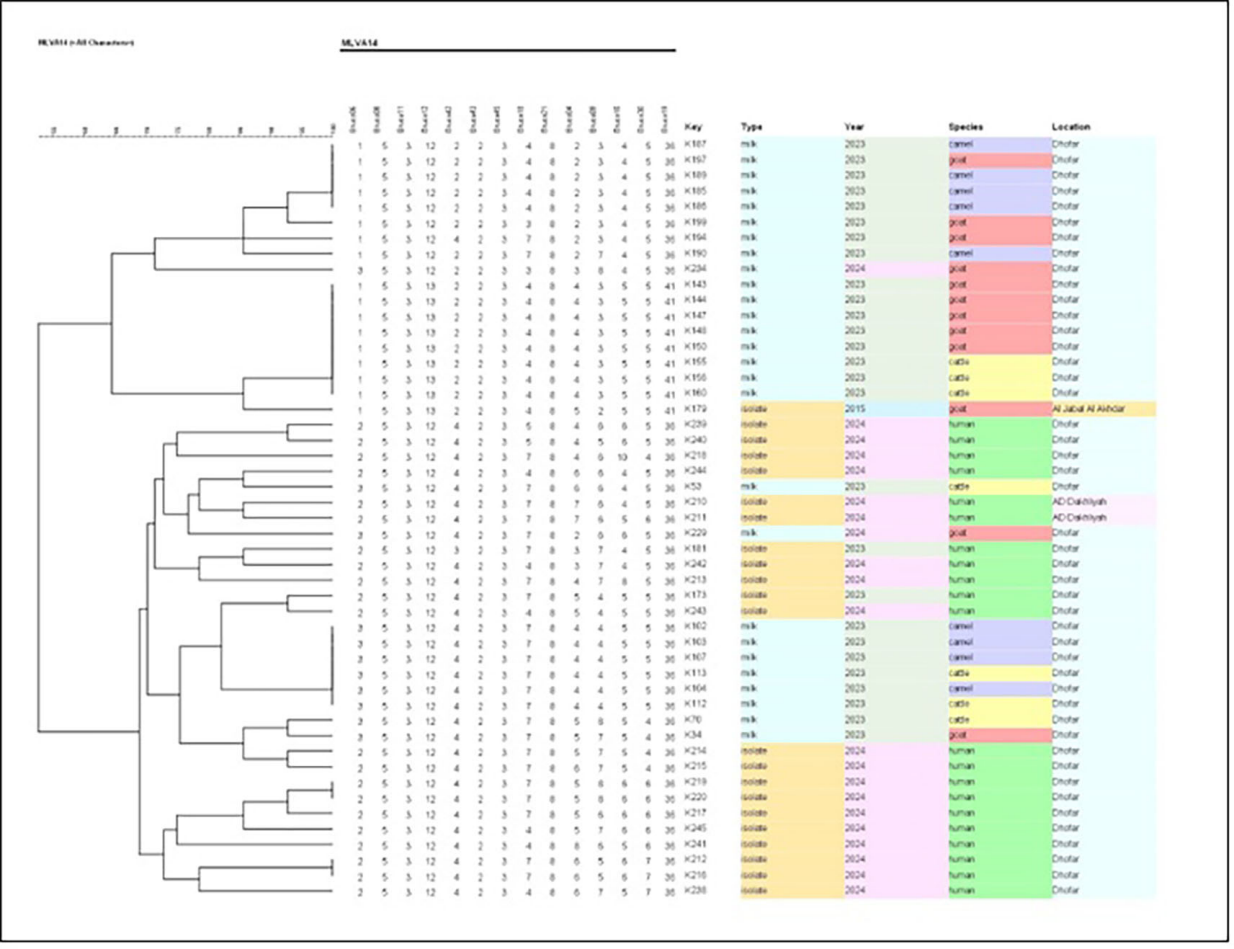
Dendrogram based on MLVA-14 genotyping using UPGMA (Unweighted Pair Group Method with Arithmetic Mean). The figure shows the genetic relationship among 49 *B. melitensis* strains recovered from human and different animal species in Oman. The columns represent the MLVA-14 profile, key (identification number), sample type, collection year, species, and geographic origin respectively.

Peaks corresponding to all 16 markers were detected in all isolates (human and goat) by capillary electrophoresis. However, only 12 out of 42 milk samples showed peaks for all 16 markers. The remaining samples lacked at least one locus. Therefore, MLVA-14 was selected as a typing approach since it allowed the inclusion of samples missing up to two loci, while excluding those missing three or more loci. As a result, 27/42 milk samples were amplified for all 14 markers (Bruce8, Bruce11, Bruce43, Bruce45, Bruce21, Bruce6, Bruce42, Bruce18, Bruce4, Bruce9, Bruce16, Bruce30, Bruce19). No sample showed multiple alleles at any locus. MLVA-14 genotyping of 49 *B. melitensis* strains showed that Bruce8, Bruce11, Bruce43, Bruce45 and Bruce21 loci were homogenous. Conversely, the most discriminatory loci were Bruce6, Bruce42, Bruce18, Bruce4, Bruce9, Bruce16, Bruce30 and Bruce19 ( Table 1). Subsequent cluster analysis allowed to recognize two main *B. melitensis* clusters including 31 different MLVA-14 genotypes ( Figure 1). The distances between strains within and between clusters were calculated based on the number of matching and differing VNTRs. In Dhofar governorate, *B. melitensis* strains extracted from camel milk (K187, K189, K185, K188) and goat milk (K197) in 2023 shared the same genotype. Moreover, *B. melitensis* strains extracted from goat milk (K43, K44, K47, K48, K50) had the same genotype as strains recovered from cattle milk (K55, K56, K60). In addition, strains retrieved from camel milk (K102, K103, K104, K107) shared the same genotype as strains from cattle milk (K112, K113). On the other hand, two human isolates (K219, K220) dating 2024 had the same genotype; also, the other two human isolates (K212, K216), identified in the same year, shared a unique genotype. The rest of the strains showed different genotypes thus forming subclusters ( Figure 1).

**Table 1.** Genetic diversity of 49 *B. melitensis* strains based on MLVA-14 genotyping.

Locus Number	Number of alleles per locus	Number of copies of tandem repeats in locus
Bruce 30	4	4, 5, 6, 7
Bruce 19	2	36, 41
Bruce 42	3	2, 3, 4
Bruce 18	4	3, 4, 5, 7
Bruce 43	1	2
Bruce 21	1	8
Bruce 09	7	2, 3, 4, 5, 6, 7, 8
Bruce 12	2	12, 13
Bruce 06	3	1, 2, 3
Bruce 08	1	5
Bruce 16	5	4, 5, 6, 8, 10
Bruce 04	7	2, 3, 4, 5, 6, 7, 8
Bruce 45	1	3
Bruce 11	1	3

**Figure 2.**
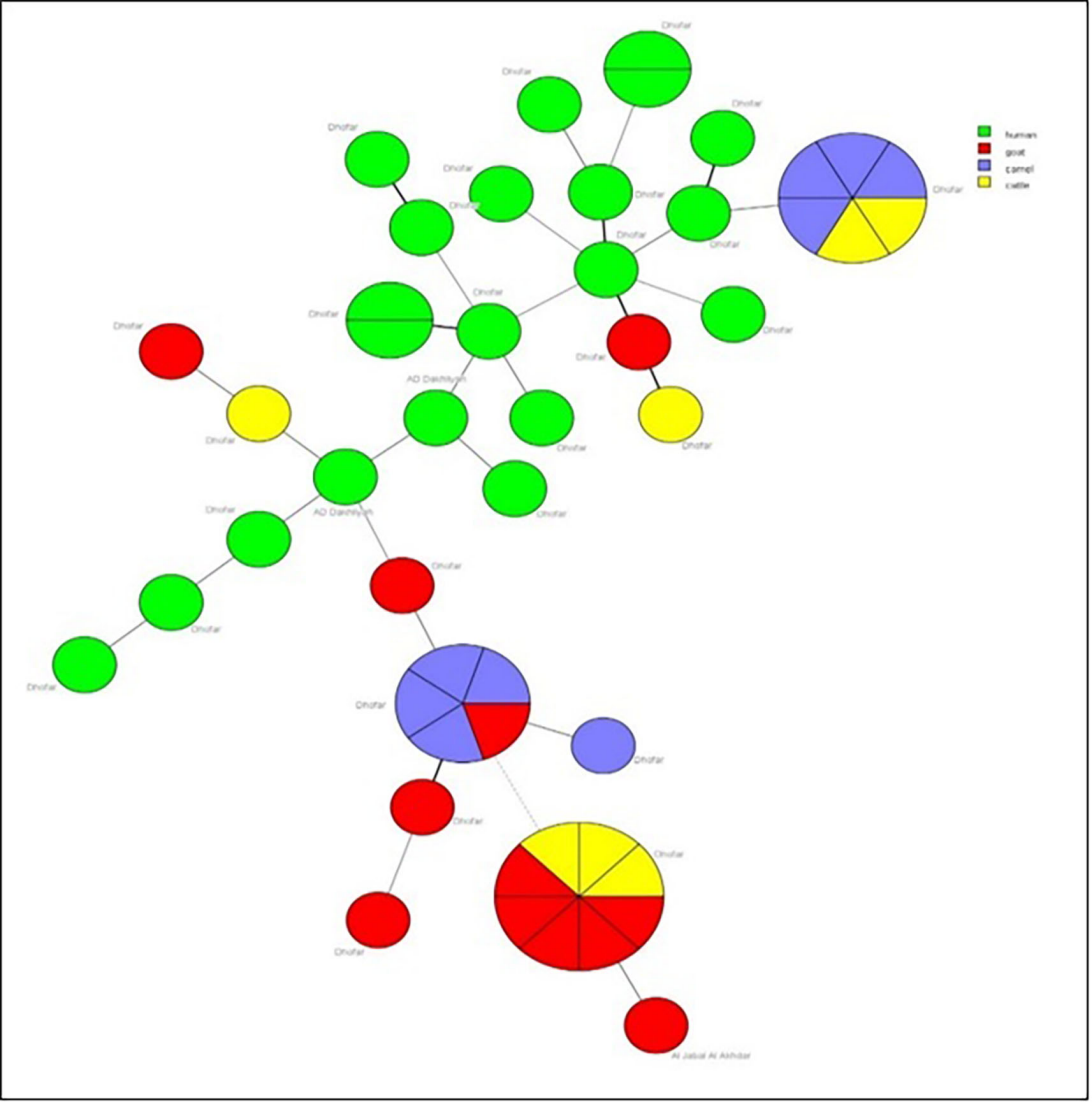
MLVA-14 Minimum Spanning Tree describing the relationships of 49 *B. melitensis* strains based on species and location. Circles represent MLVA-14 genotypes, colored according to the species, and the size of the circle indicates the number of strains within that genotype *.*

**Figure 3.**
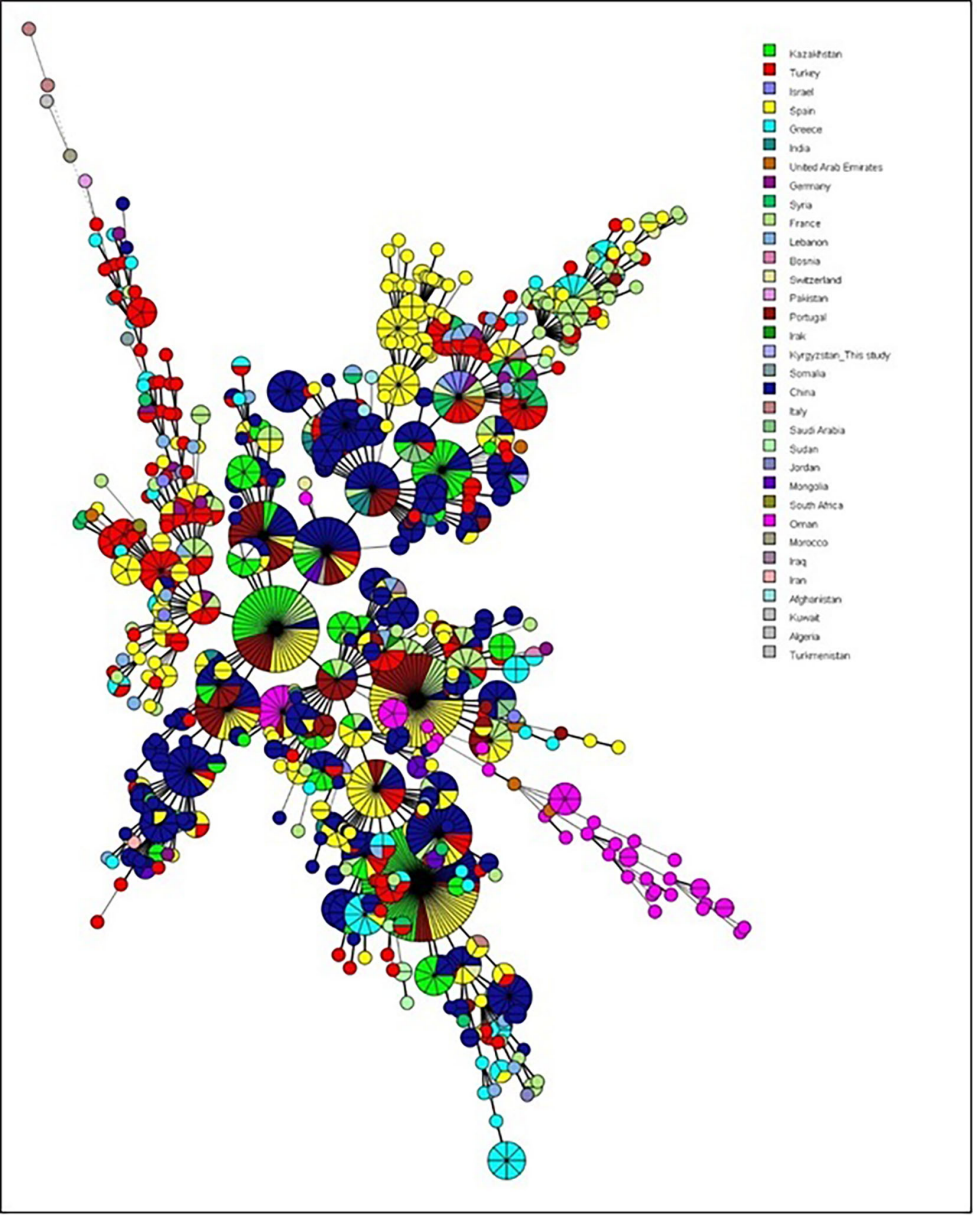
MLVA-14 Minimum Spanning Tree describing the relationships of 49 *B. melitensis* isolates with worldwide isolates. Circles represent MLVA-14 genotypes, colored according to the country of origin, and the size of the circle indicates the number of strains within that genotype.

**Figure 4.**
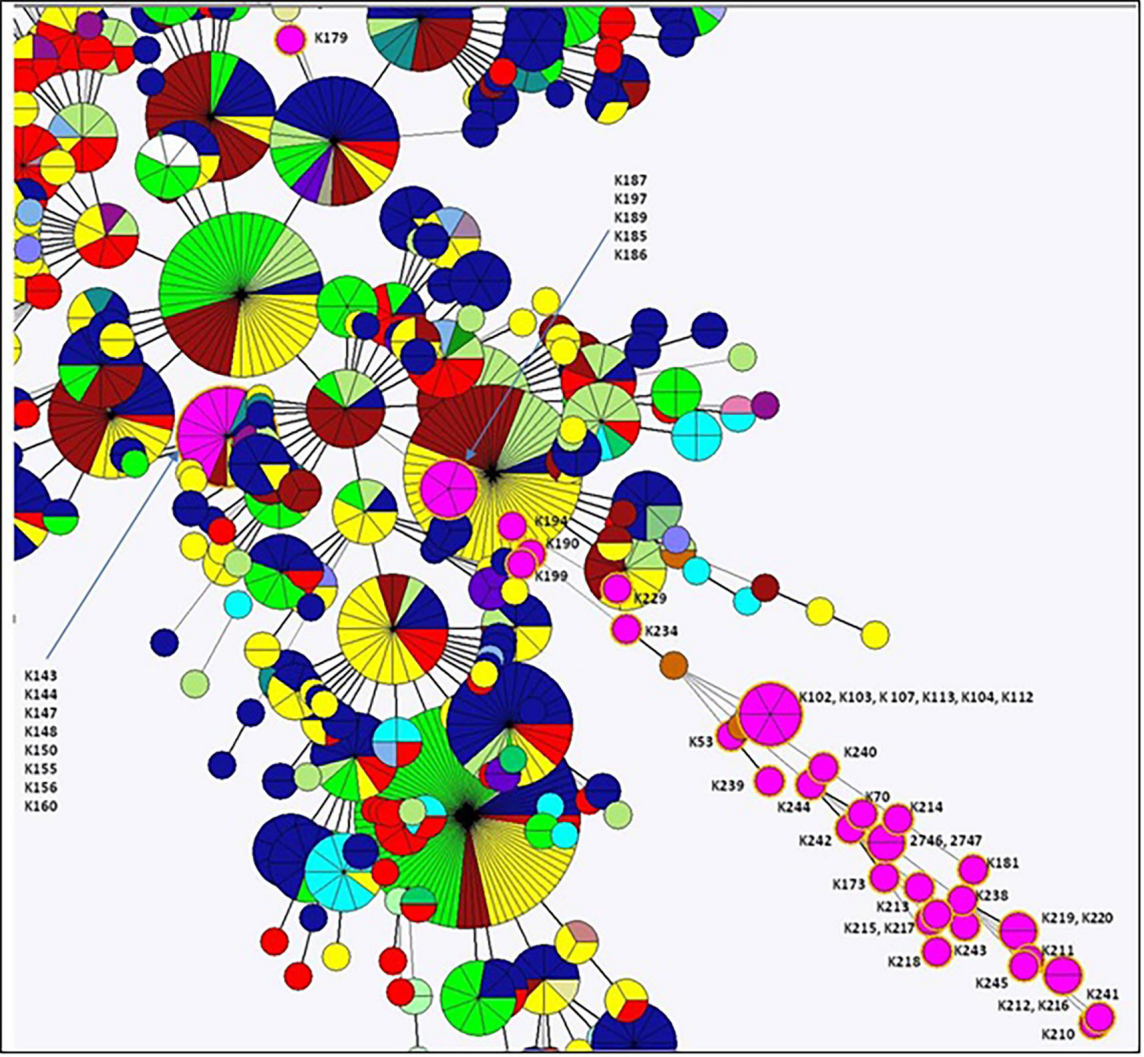
MLVA-14 Minimum Spanning Tree (zoomed) describing the relationships of 49 *B. melitensis* isolates with worldwide isolates with strains IDs. Circles represent MLVA-14 genotypes, colored according to the country of origin, and the size of the circle indicates the number of strains within that genotype.

Minimum spanning trees (MST) were generated for the 49 Omani strains based on host species and geographical location ( Figure 2). MST presents the genetic profiles, with circles representing individual or grouped strains and edges reflecting genetic distances. Circle colors indicate the host; green (human), red (goat), purple (camel), and yellow (cattle), while circle sizes correspond to the number of isolates sharing an identical genetic profile. Human isolates (green) were related, suggesting a shared genetic background. *Brucella* strains extracted from goat milk (red) showed distinct clustering, with some overlap with strains extracted from camel milk (purple) and cattle milk (yellow). For geographic annotations, most strains originated from Dhofar Governorate, two human isolates from AD Dakheliya Governorate and a goat isolate from Al Jabal Al Akhdhar which located in AD Dakheliya Governorate. The phylogeographic patterns of the studied *Brucella* strains were compared to MLVA profiles available in the international database ( Figure 3 & Figure 4). It should be noted that for MST worldwide analysis, Bruce19 locus was not included since many foreign isolates were not typed for this marker and to avoid numbering uncertainty due to the recent discovery of rare alleles at the Bruce19 locus that changed the nomenclature. ^11^ The Omani strains belong to the East Mediterranean lineage. In the MST analysis with worldwide isolates, most Omani strains tend to separate forming a country branch, which includes two strains from the United Arab Emirates (in brown). However, another group of Oman strains clusters with Spain, Portugal, India, Turkey, and China strains. These two Omani clusters are connected to a junction node that includes isolates from Portugal, China, France and Kazakhstan. Finally, it can be noticed one “outlier” Oman strain correlated with a big cluster mostly formed by isolates from China, but also including strains from Portugal, Kazakhstan, Turkey, Mongolia, Marocco, France and Spain.

## Discussion

This study aimed to genotype *Brucella* strains circulating in human and livestock in Oman. To confirm the presence of *Brucella* in milk samples, a milk ring and rapid tests were used. Then, real-time PCR was also carried out, followed by conventional PCR based on three *Brucella*-specific primer pairs. Moreover, species-specific primers (Bruce-ladder) were employed, as described by Ref. 21.

This study, to the best of our knowledge, is the first to use DNA extracted directly from milk samples for MLVA analysis. This approach likely explains the missing loci in the PCR products of some samples. Moreover, extraction of DNA directly from milk may yield low-quality DNA. Also, the presence of PCR inhibitors in milk may hinder PCR reactions. In general, it should be taken into consideration that milk samples might contain more than one *Brucella* isolates, and multiple alleles can be amplified at certain loci thus hampering inference of MLVA haplotypes. Such occurrence seems to be unlikely given the fact that, in this study, all milk samples investigated were infected by a single strain; however, the possibility to detect co-infections with multiple strains would obviously increase when working with bulk milk samples in endemic areas. Nonetheless, we decided to work with milk samples to avoid the risk associated with culturing *Brucella.* Moreover, the lack of a biosafety level 3 cabinet (BSL-3) at the microbiology laboratory in the animal and veterinary sciences department in SQU was an obstacle to dealing with the zoonotic nature of *Brucella.* It appears that it is worth mentioning that all human isolates used in this study were cultured at Sultan Qaboos University Hospital (SQUH) and Sultan Qaboos Hospital in Salalah (SQH). All these isolates were successfully typed at each locus for the 16 markers. Therefore, isolates remain the best choice for MLVA analysis. Interestingly, even a study by Ref. 31 using isolates had reported missing loci for some markers, such as Bruce07 and Bruce19, underscoring potential challenges in MLVA analysis.

Consequently, MLVA-14 analysis was utilized to genotype 49 *B. melitensis* strains, clustered into two main groups comprising 31 distinct genotypes. Based on the dendrogram **(** Figure 1) and ( Table 1), homogeneity was observed in Bruce8, Bruce11, Bruce43, Bruce45, and Bruce21 loci with a monomorphic profile in other studies. ^7, 32^ On the other hand, Bruce 21 presented variable allelic types in previous studies. ^7, 11, 31, 33^ In this study, Bruce6, Bruce42, Bruce12, and Bruce19 loci were moderately variable with two to three allelic types, also moderately variable in other studies. ^11, 30^ The highly discriminatory markers in this study were found in Bruce4, Bruce9, Bruce16, Bruce18, and Bruce30. Particularly, Bruce4 and Bruce9 exhibited diverse allelic types (7 types), contributing significantly to the differentiation between *Brucella* strains. In a study by Tiller et al. (2019), Bruce4, Bruce9, Bruce16, and Bruce18 were also polymorphic, whereas Bruce 30 tended to be more conserved. ^32^ Kulakov et al. (2011) reported that Bruce4 and Bruce16 were highly polymorphic, whereas Bruce9 and Bruce18 were monomorphic. ^34^ In another study, Bruce4, Bruce30, and Bruce16 were discriminatory markers. ^11^

Overall, the clustering patterns and genotypic differences or similarities observed in this study align with findings from previous research, emphasizing the usefulness of MLVA in studying *Brucella* genetic diversity and epidemiology.

The genetic similarity observed among *B. melitensis* strains from camel milk (K187, K189, K185, K188) and goat milk (K197) samples in Dhofar Governorate suggests potential shared reservoirs or transmission pathways within livestock populations in the region. Similarly, the identical genotypes identified in goat milk strains (K43, K44, K47, K48, K50) and cattle milk strains (K55, K56, K60) highlight the interconnected nature of *Brucella* transmission across different animal hosts, possibly through shared environments, grazing lands, or interspecies interactions. Furthermore, the shared genotypes between camel milk strains (K102, K103, K104, K107) and cattle milk strains (K112, K113) reinforce the role of camels and cattle as significant reservoirs for *B. melitensis* in Dhofar Governorate. The transmission of *B. melitensis* between different animal species was reported previously in Oman. ^4, 5, 35– 37^ Moreover, human isolates K219 and K220 shared the same genotype, while K212 and K216 formed another group with identical genotypes. These findings are consistent with previous studies which indicate that human infection is often linked to livestock reservoirs due to zoonotic transmission, frequently through the consumption of unpasteurized dairy products or direct contact with infected animals. ^36, 38– 41^ In addition, the majority of human cases in this study reported a history of consuming unpasteurized raw milk across all age groups according to SQUH and SQH. Notably, the highest number of cases was observed among children under the age of ten years. However, that might be because of their developing immune systems, making them more susceptible to infections like *Brucella.* Additionally, children are more likely to consume unpasteurized milk, especially in regions where it is considered a traditional dietary essential. Limited awareness of the risks associated with raw milk consumption, combined with potential exposure to contaminated environments, such as farms or infected animals, further increases their risk. Advancement of the healthcare system in Oman has led to improved diagnosis and reporting of illnesses across all age groups.

The genetic heterogeneity of other strains, divided into subclusters, reflects the diverse *Brucella* population in Dhofar Governorate, highlighting the need for localized control measures and management procedures. ^4, 36, 37^ Our MLVA analysis provides valuable insights also into the epidemiology of *Brucella* in Oman, including host specificity, geographic clustering, and potential transmission dynamics.

The phylogeographic analysis of 49 *Brucella melitensis* strains from Oman was compared to international MLVA-15 profiles as previously described. ^7, 30, 33^ This provides significant insights into the genetic relationships and potential transmission patterns of these strains worldwide. In MST analysis integrating worldwide isolates, most Omani strains formed a distinct country-specific branch, with close association with two strains from the United Arab Emirates. Specifically, this branch included strains recovered from human (K234, K239, K244), cattle and camel milk (K53, K70, K102, K103, K107, K104, K112, K113). This finding suggests a localized genetic lineage, likely shaped by regional factors such as shared livestock trade routes or common environmental reservoirs in the Arabian Peninsula. ^4, 42^ Additionally, another cluster of Omani strains recovered from goat and cattle milk (K143, K144, K147, K148, k150, K155, K156, K160) grouped with isolates from Spain, Portugal, India, Turkey, and China. This broader clustering indicates potential historical or trade-driven links, reflecting the global trade of livestock or animal products, which has likely influenced the genetic diversity of *B. melitensis* strains. ^35, 36^ Interestingly, one “outlier” Omani goat isolate (K179) was closely related to a cluster including mainly Chinese isolates, but also isolates from countries worldwide (i.e., Portugal, Kazakhstan, Mongolia, Turkey, Spain, Morocco, France) highlighting an exception to the broader clustering trends. This unique pattern may represent a rare introduction of a strain through trade, livestock importation, and, or human travel. It appears that the complexity of the trading economy may explain some of the observed genetic relationships; for example, Oman imports dairy products, eggs, honey, and other edible products from Kazakhstan.

These results underscore the complexity of *Brucella* phylogeography, reflecting both regional and global transmission dynamics. The distinct clustering of most Omani strains emphasizes the potential role of localized environmental and epidemiological factors in shaping *Brucella* diversity. However, the observed connections with strains from other countries emphasize the importance of monitoring international trade and the movement of livestock to prevent the spreading of brucellosis. ^43^

## Conclusions

This research is the first to genotype, compare and correlate *B. melitensis* strains from humans and livestock in Oman, through MLVA-14. The results illustrate a genetic relationship between strains of human and livestock origin, further illustrating zoonotic transmission and making these populations interconnected. Also, the association of the genotypes and their distribution among different animal species corroborates the established transmission routes among livestock in Dhofar and AD Dakhiliya Governorates. Isolates produced relatively better genotyping than milk samples, thus demonstrating the need for careful and proper sample selection and handling. Building on these results, however, there is a need for a One Health approach involving both the veterinary and the public health sectors in brucellosis control efforts. Major next steps include broadening the sampling base from other governorates in Oman, implementing whole genome sequencing together with MLVA for even more comparative studies, and developing a national *Brucella* surveillance strategy that incorporates a centralized genotype repository. Finally, strengthening laboratory capacities through expertise and equipment for *Brucella* isolation and characterization would improve the surveillance system and disease management in Oman.

This study was approved by the Medical Research Ethics Committee (MERC) in the College of Medicine & Health Sciences at Sultan Qaboos University (SQU) REF. NO. SQU-EC/060/2023 for using human samples. The research project submitted to the Medical Research Ethics Committee (MREC), College of Medicine and Health Sciences, Sultan Qaboos University for re-consideration and approval was discussed. The modifications received by the Committee on 3rd May 2023 in response to the comments raise during the 30th March 2023 meeting were found to be satisfactory. The Committee has considered the research project acceptable and therefore approval was granted. For animal samples, there was no need for the Sultan Qaboos University ethical committee’s approval as the study did not involve experimental research on animals. The animal milk samples were submitted to the central laboratory for routine diagnosis and a verbal consent was granted to use the samples for our project.

### Consent to participants

All participants gave an oral consent to participate in this research work.

## Animal ethics

The experiment was conducted in Dhofar and AD Dakhiliyah governorates, Oman, for milk samples. The samples were collected from grazing animals in the open, owned by private individuals with their consent and guidance, who managed animal husbandry as per rural tradition. There was minimal interaction with the animals for the collection of milk for further investigation. Minimal animal manipulation (handling &amp; restraint) was observed for milking by experienced personnel, ensuring an aseptic collection procedure. The sample size was much smaller than the normal milking volume for each animal.

The animals were needed only for milk samples. The milking was done in a clean quiet area to ensure a peaceful environment for the animals. There was minimal handling and restraint under the consent, help, and guidance of the owners who are very familiar with their stock. Milking was carried out aseptically by experienced technicians. The clinician was around to ensure the safety of the animals during the procedure. This asserts that animal welfare was not compromised in any way more than minimal normal handling for aseptic milking under clinical supervision in the presence of animal owners.

## Data Availability

Figshare: Genetic diversity of brucella melitensis in Oman, https://doi.org/10.6084/m9.figshare.28190633. ^44^ Title: Paper-Raw Data.zip Author: Yasmin Ahmed Description: Genetic diversity of brucella melitensis in Oman It contains three files
1. FSA files contain the Capillary electrophoresis raw data 2. An excel file contains comparison between Oman strains & worldwide strains 3. A word document file with detailed description of human & animal samples used in the study. Names of human patients were not revealed. Data are available under the terms of the Creative Commons Attribution 4.0 International license (CC-BY 4.0).
