# Supplementary material for: East Mediterranean Lineage of Brucella melitensis in Human Isolates and Milk Samples in Oman Using MLVA-14
Source: F1000Res. 2026 Jun 1;14:191. Originally published 2025 Feb 12. [Version 3] doi: 10.12688/f1000research.161111.3 (PMC13294665; doi:10.12688/f1000research.161111.3)
Supplement: Peer review history

# Reviewer report: Reviewer response for version 3

**DOI:** 10.5256/f1000research.201344.r491664

**Published:** 2026-06-24

**Authors:** Adel MAMMERI

**Affiliations:** 1 Department of Veterinary Sciences, Faculty of Sciences, University of M’sila, M’Sila, Algeria

I have some minor reservations :

**Page 4, line 3 (Introduction)** : You reported in the last paragraph of the introduction that no previous studies in Oman have used MLVA to analyze the genetic diversity of Brucella melitensis in both human and milk samples, limiting understanding of transmission and control.

 However, I found several studies in the same context. As an example, this is one example of studies who used MLVA for B. melitensis in Oman :

 Foster JT, Walker FM, Rannals BD, Hussain MH, Drees KP, Tiller RV, Hoffmaster AR, Al-Rawahi A, Keim P and Saqib M (2018) African Lineage Brucella melitensis Isolates from Omani Livestock. Front. Microbiol. 8:2702. doi: 10.3389/fmicb.2017.02702.

 So please, confirm that your statement is true.

**Page 4, line 6  (Introduction):** There is a repetition in the paragraph situated in the end of introduction : This study addresses this gap by investigating the genetic makeup of **Brucella strains Brucella strains** circulating in different geographical locations in Oman in both human and livestock using MLVA.

**Page 6, lines 3 and 4 (Results):** it lacks Bruce 12 in the brackets (Bruce8, Bruce11, Bruce43, Bruce45, Bruce21, Bruce6, Bruce42, Bruce18, Bruce4, Bruce9, Bruce16, Bruce30, Bruce19).

**Page 8, line 5 (Discussion) :** Please, confirm the basis of this statement, by searching rigorously in the former studies :This study, to the best of our knowledge, is the first to use DNA extracted directly from milk samples for MLVA analysis.

**Page 10, line 24 (Discussion) :** The transmission of B. melitensis between different animal species was reported previously in Oman. DThis statement should be consolidated by one referenced paragraph of about 5-8 lines to explain the the impact of mixed-species livestock farming (often unregulated) on the persistence and transmission of B. melitensis.

**Page 11, line 14 (Conclusions) :** Please, confirm the basis of this statement, by searching rigorously in the former studies :This study represents the first comprehensive genotyping and comparative analysis of B. melitensis strains from humans and livestock in Oman using MLVA-14.

Is the work clearly and accurately presented and does it cite the current literature?

Yes

If applicable, is the statistical analysis and its interpretation appropriate?

Yes

Are all the source data underlying the results available to ensure full reproducibility?

Yes

Is the study design appropriate and is the work technically sound?

Yes

Are the conclusions drawn adequately supported by the results?

Yes

Are sufficient details of methods and analysis provided to allow replication by others?

Yes

Reviewer Expertise:

Veterinary epidemiology, zoonoses, brucellosis, animal welfare and health.

I confirm that I have read this submission and believe that I have an appropriate level of expertise to confirm that it is of an acceptable scientific standard.

# Reviewer report: Reviewer response for version 2

**DOI:** 10.5256/f1000research.196492.r469956

**Published:** 2026-04-10

**Authors:** AbdulHamid Settenda Lukambagire

**Affiliations:** 1 Kilimanjaro Clinical Research Institute, Moshi, Tanzania

I feel there is too much detail in the ethical considerations, for instance this statement is unnecessary ... "The modifications received by the Committee on 3rd May 2023 in response to the comments raised during the 30th March 2023 meeting were found to be satisfactory. The Committee has considered the research project acceptable and therefore approval was granted."

 For Fig 1., although this diagram is rather informative, I would propose that the authors redesign it as a flow diagram/ step-wise diagram.

 It is rather difficult to follow the source, tests and samples highlighted therein.

 Also, since the authors have elected to include all the series of tests conducted on samples in this figure, it might be useful to also to provide a table/ summary of the various sample types and results (even as  supplementary material).

 Also, how did the authors account for the potential low DNA yield from direct milk extraction to allow MLVA typing?

 A map to show the relative locations of the sample sources would be useful for readers not familiar with the geography of the country or region in general.

 I found the discussion points quite well detailed and compared with previous work. However, I found it very difficult to distill the point each of these paragraphs was trying to put across. In the first paragraph of the discussion, authors mention multiple points, most of which actually sound like limitations to the study findings/ inferences. I would propose these are clearly stated in a final paragraph before conclusions.

 The second paragraph also discusses a lot about genotypes and loci...but doesn't clearly communicate a take home message from this?! I would presume that two core clusters (with various loci) were observed...one fairly conserved, as observed in previous studies, and another quite variable. All these points are critical to the overall understanding of the work done, but I feel the authors have left these deductions to the discernment of the reader, which is rather self-defeating.

 It all leads to a conclusion that is not very well appreciated given the great study design and results presented. I would recommend the discussion section is re-written to provide succinct take-home language.

 Lastly, there is a lot of repeated ethical language abutted to the conclusion paragraph. Since most of this language is repeated from the methods section, this should either be removed completely, or rewritten and summarized under an ethics statement (review journal format for this).

Is the work clearly and accurately presented and does it cite the current literature?

Yes

If applicable, is the statistical analysis and its interpretation appropriate?

Yes

Are all the source data underlying the results available to ensure full reproducibility?

Yes

Is the study design appropriate and is the work technically sound?

Yes

Are the conclusions drawn adequately supported by the results?

Yes

Are sufficient details of methods and analysis provided to allow replication by others?

Yes

Reviewer Expertise:

Brucella (zoonotic disease) diagnosis, molecular epidemiology, immunology; Medical parasitology

I confirm that I have read this submission and believe that I have an appropriate level of expertise to confirm that it is of an acceptable scientific standard, however I have significant reservations, as outlined above.

# Response

**Published:** 2026-05-10

**Authors:** Yasmin ElTahir

**Affiliations:** Animal & Veterinary Sciences, Sultan Qaboos University, Muscat, Muscat Governorate, Oman

**Reviewer comment:**

 I feel there is too much detail in the ethical considerations, for instance this statement is unnecessary ... "The modifications received by the Committee on 3rd May 2023 in response to the comments raised during the 30th March 2023 meeting were found to be satisfactory. The Committee has considered the research project acceptable and therefore approval was granted."

**Response:**

 We thank the reviewer for this observation. We have shortened the ethical considerations section by removing overly detailed procedural descriptions (e.g., internal committee review steps) and retained only the essential information required by the journal format. In addition, redundant ethical statements previously repeated in the Conclusions have been removed and consolidated into a concise Ethics Statement. Reviewer comment:

**Reviewer comment:**

 For Fig 1… redesign as a flow diagram… difficult to follow

**Response:**

 We agree with the reviewer. Figure 1 has been redesigned as a clearer step-wise workflow diagram to improve readability and better represent the sequence of sampling, testing, and MLVA analysis.

**Reviewer comment:**

 Provide a table/summary of sample types and results

**Response:**

 We thank the reviewer for this suggestion. A summary table describing the different sample types, number of samples, and key results has been added as Supplementary Material (Table S1).

**Reviewer comment:**

 How did the authors account for low DNA yield from milk?

**Response:**

 We thank the reviewer for this important point. We have clarified this aspect in the Discussion section. In particular, we highlight that:

 (i) direct DNA extraction from milk may result in lower DNA yield and quality,

 (ii) the presence of PCR inhibitors in milk can affect amplification efficiency, and

 (iii) this limitation contributed to missing loci in some samples, which led us to adopt MLVA-14 instead of MLVA-16.

 These limitations are now explicitly discussed.

**Reviewer comment:**

 A map of sampling locations would be useful

**Response:**

 We agree with the reviewer. A map showing the sampling locations has been added as a new figure (Figure X / Supplementary Figure S1) to provide geographic context.

**Reviewer comment:**

 Discussion difficult to follow; unclear take-home messages

**Response:**

 We thank the reviewer for this insightful comment. The Discussion has been substantially revised to improve clarity and structure. In particular:

- key findings are now explicitly stated at the beginning of each paragraph;
- a clear “take-home message” has been added regarding the identification of two main clusters (one relatively conserved and one more variable);
- a dedicated paragraph summarizing study limitations has been introduced before the Conclusions.

**Reviewer comment:**

 Ethical language repeated in conclusions

**Response:**

 We agree with the reviewer. Redundant ethical statements have been removed from the Conclusions and replaced with a concise Ethics Statement in accordance with the journal format.

# Reviewer report: Reviewer response for version 1

**DOI:** 10.5256/f1000research.177103.r388868

**Published:** 2025-06-14

**Authors:** Md. Sadequl Islam

**Affiliations:** 1 Hajee Mohammad Danesh Science and Technology University, Dinajpur, Bangladesh

**Introduction**

**Critique:**

1. **Clarity and Focus:** The introduction provides a broad overview of brucellosis and its zoonotic importance. However, it lacks a sharply focused research gap or hypothesis. The shift from general background to specific study objectives is gradual and could be more clearly demarcated.
2. **Redundancy:** Multiple references to Brucella species and diagnostic tools make the narrative somewhat repetitive (e.g., PCR methods and serological tests are listed in detail without always linking back to the rationale for MLVA-14).
3. **Overload of Technical Details:** The excessive explanation of general diagnostic methods (e.g., Rose Bengal, ELISA) dilutes the focus from the genetic diversity angle, which is the paper’s strength.

**Suggestions for Improvement:**

- Explicitly highlight the research gap early (e.g., “No previous studies in Oman have analyzed the genetic diversity of *B. melitensis* using MLVA in both human and milk samples”).
- Trim redundant or overly detailed background information not directly tied to the study’s rationale.
- Consider ending the introduction with a concise, hypothesis-driven objective statement.

**Methods**

**Critique:**

1. **Strength:** Well-detailed methodology, especially ethical approvals and sample handling—commendable from a biosafety and animal ethics perspective.
2. **Lack of Clarity in MLVA Details:** Though the MLVA-16 and MLVA-14 transition is mentioned, the justification for reducing the number of markers (due to missing loci) could be clarified earlier.
3. **Reproducibility Gap:** DNA quality control metrics and reasons for PCR failure in some samples could have been explained more robustly.

**Suggestions for Improvement:**

- Include DNA quality thresholds or quantification methods.
- Add a flow diagram summarizing the sample collection, screening, and selection for final MLVA analysis.

**Results**

**Suggestions for Improvement:**

- Include a map showing sampling locations for geographic context.
- The study presents valuable MLVA-14 genotyping data on *Brucella melitensis* strains from Oman, contributing to the regional and global understanding of Brucella epidemiology. However, the manuscript does not indicate whether these genotypes have been submitted to the international MLVA Bank ( http://mlva.u-psud.fr/brucella/). I strongly recommend that the authors deposit the MLVA profiles in this publicly accessible database. Doing so will enhance the transparency, reproducibility, and comparative utility of the data for future studies and global surveillance efforts. Please include a statement confirming submission in the Methods or Data Availability section.

**Discussion**

**Suggestions for Improvement:**

- Consider including confidence metrics (e.g., bootstrap values) for MST branches to strengthen phylogeographic conclusions.
- Reframe speculative claims as hypotheses needing further exploration unless supported by trade/import data.
- Discuss limitations such as potential bias in sample collection (mostly Dhofar) and the use of milk vs isolate DNA.
- **After the sentence in the discussion section please add the citation below “Overall, the clustering patterns and genotypic differences or similarities observed in this study align with findings from previous research, emphasizing the usefulness of MLVA in studying Brucella genetic diversity and epidemiology.”**

* *
*“Overall, the clustering patterns and genotypic differences or similarities observed in this study align with findings from previous research, emphasizing the usefulness of MLVA in studying Brucella genetic diversity and epidemiology. A similar molecular study conducted in Bangladesh reported the first genetic characterization of Brucella abortus biovar 3 using MLVA, reinforcing the value of this method in endemic regions for epidemiological insight and disease control planning (Islam et al., 2019).”*

**Citation:**

 Islam, M. S., Garofolo, G., Sacchini, L., Dainty, A. C., Khatun, M. M., Saha, S., & Islam, M. A. (2019). First isolation, identification, and genetic characterization of *Brucella abortus* biovar 3 from dairy cattle in Bangladesh. *Veterinary Medicine and Science, 5*(4), 556–562. (refer to 1 )This addition provides a strong, real-world example from a comparable endemic region.

**Conclusion**

**Critique:**

1. **Overstated Generalization:** The assertion that this is the “first study” using milk DNA in MLVA analysis may require qualification or citation.
2. **Missing Specific Recommendations:** The section could benefit from more actionable public health recommendations.

**Suggestions for Improvement:**

- Suggest policy-level implementations (e.g., pasteurization campaigns, livestock vaccination).
- Recommend steps for improving laboratory infrastructure based on study constraints.

Is the work clearly and accurately presented and does it cite the current literature?

Yes

If applicable, is the statistical analysis and its interpretation appropriate?

Not applicable

Are all the source data underlying the results available to ensure full reproducibility?

Yes

Is the study design appropriate and is the work technically sound?

Yes

Are the conclusions drawn adequately supported by the results?

Yes

Are sufficient details of methods and analysis provided to allow replication by others?

Partly

Reviewer Expertise:

Microbiology, Molecular biology, Enviromental toxicology, Gene expression

I confirm that I have read this submission and believe that I have an appropriate level of expertise to confirm that it is of an acceptable scientific standard, however I have significant reservations, as outlined above.

## References

[1] Islam, M. S., Garofolo, G., Sacchini, L., Dainty, A. C., Khatun, M. M., Saha, S., & Islam, M. A. (2019). First isolation, identification, and genetic characterization of Brucella abortus biovar 3 from dairy cattle in Bangladesh. Veterinary Medicine and Science, 5(4), 556–562. https://doi.org/10.1002/vms3.511.10.1002/vms3.193PMC686845231452358

[2] : Extract of neem (Azadirachta indica ) leaf exhibits bactericidal effect against multidrug resistant pathogenic bacteria of poultry. *Veterinary Medicine and Science* .2021;7(5) : 10.1002/vms3.511 1921-1927 10.1002/vms3.511 PMC846424833955693
